# Exploring the Status of Preference, Utilization Practices, and Challenges to Consumption of Amaranth in Kenya and Tanzania

**DOI:** 10.1155/2022/2240724

**Published:** 2022-06-18

**Authors:** Winnie A. Nyonje, Ray-Yu Yang, Dyness Kejo, Anselimo O. Makokha, Willis O. Owino, Mary O. Abukutsa-Onyango

**Affiliations:** ^1^School of Food and Nutritional Sciences, Jomo Kenyatta University of Agriculture and Technology, Nairobi, Kenya; ^2^World Vegetable Centre, Tainan, Taiwan; ^3^Food and Fertilizer Technology Center for the Asian and Pacific Region, Taipei, Taiwan; ^4^World Vegetable Centre - Eastern & Southern Africa, Arusha, Tanzania; ^5^Department of Horticulture, Jomo Kenyatta University of Agriculture and Technology, Nairobi, Kenya

## Abstract

African leafy vegetables such as amaranth have been utilized since time immemorial both as food and as medicine. These vegetables grew naturally in most rural environments, but currently most of them are cultivated both for home consumption and for sale. The aim of this study was to identify the most preferred amaranth species and cooking and utilization practices, as well as the beliefs and attitudes that encourage or discourage use of this vegetable. The study was carried out in seven counties of Kenya and in three regions in Tanzania. Twenty Focus Group Discussions (FGDs) with members of the community and twenty Key Informant Interviews (KIIs) with agricultural and nutrition officers were conducted in the study areas to obtain information on preferred varieties, sources of amaranth vegetables, common cooking methods, alternative uses, beliefs and taboos surrounding amaranth consumption, and the challenges experienced in production and consumption. The findings of the study showed that amaranth is one of the most commonly consumed indigenous vegetables in Kenya and Tanzania. The preference for varieties and cooking habits differs depending on the community and individuals. *Amaranthus dubius* and *Amaranthus blitum* were most common in Kenya, while *Amaranthus dubius* and *Amaranthus hypochondriacus* were most common in Tanzania. Most people consumed these vegetables because they were affordable and available or because of circumstance of lacking other foods. Regarding cooking, final taste was mostly considered rather than nutritional attribute. Several alternative uses of amaranth such as uses as medicine and livestock feed were also reported, as well as some beliefs and taboos surrounding the vegetable. Training on nutritional attributes and promotion of food preparation practices that ensure maximum nutrient benefits from amaranth is needed at the community level to realize the nutritional importance of the vegetables. Hands-on training and demonstrations were the most preferred modes of passing information.

## 1. Introduction

The amaranth vegetable (*Amaranthus* spp.) is one of the most commonly consumed African Indigenous Vegetables (AIVs). However, these vegetables have faced many years of neglect [1], with young consumers and urban dwellers equating the vegetables with traditional lifestyles [2]. All over the world, there have been notable changes in food consumption patterns, referred to as nutrition transition, as people began to adapt to new socioeconomic and environmental changes [3, 4]. This could result in either decrease or increase in consumption of certain foods such as the traditional vegetables. Demand and consumption of African indigenous vegetables such as amaranth are currently on the rise [5, 6] owing to recent research on the indigenous vegetables, which has shown that the vegetables are rich in vitamins, minerals, and bioactive compounds and are also easy to produce and cook [7]. Despite the growing demand, there has been minimal improvement in the nutrition and health of consumers as micronutrient deficiencies (hidden hunger) continue to cause poor motor and cognitive development and even lead to death [8].

Nutritional benefits from amaranth vegetable could depend on several factors, including species selected [9], production practices, and cooking method used. Certain beliefs and negative attitudes have over the years led to neglect of amaranth vegetable. Among such attitudes was the consideration of the vegetable as a weed and a poor man's food. Certain attitudes may also exist which lead to increased consumption of this vegetable; for example, spider plant (*Cleome gynandra*) is believed to be a blood booster in some areas of Kenya. There are many amaranth varieties with morphological and genetic variability, which is observed in plant characteristics such as inflorescence type, seed color, precocity, leaf color, nutritional contents, and resistance to pests and diseases [10, 11], some of which also serve as basis for selection by consumers.

The consumption of indigenous vegetables is greatly influenced by cultural backgrounds; hence, some types and varieties are associated only with specific communities [12]. Most rural communities in parts of Kenya and Tanzania are believed to be the biggest consumers of African indigenous vegetables including amaranth. A study conducted in Kenya indicated a higher consumption intensity of leafy AIVs in rural dwellers compared to urban dwellers [13]. However, preferences may vary as to types and varieties of different amaranth consumed, which could be determined by cultural and social factors. A study carried out in Tanzania showed that strong cultural food beliefs and taboos still exist among communities, which strongly influence attitudes towards traditional vegetables in general [14]. It is, however, not clear which beliefs are these and whether they negatively or positively affect consumption of amaranth.

Utilization methods of this vegetable could also differ, as well as cooking methods, which may affect the nutritional attributes of the amaranth either positively or negatively. Different communities prefer different species of the vegetables, but specific species for the different regions are not known. Factors including unfamiliar taste, small leaf sizes, low shelf life, and seasonal availability were reported to contribute to low consumption of indigenous vegetables including amaranth in various populations in Kenya [15].

Cooking vegetables improve edibility and also induce significant changes in physical characteristics, chemical composition, biological characteristics, and bioavailability of vegetable nutritional components. Most leafy vegetables like amaranth are cooked prior to consumption based on convenience and taste preferences rather than retention of nutrients and health promoting compounds [16]. Cooking vegetables destroys microorganisms and reduces antinutrients, thereby increasing the safety; it also enhances digestibility and bioavailability of nutrients. Cooking methods and duration of cooking of amaranth vegetables, as well as added ingredients, vary widely in communities. In Western Kenya, for instance, amaranth is mostly cooked in combination with other vegetables including spider plant (*Cleome gynandra*) and nightshade (*Solanum scabrum/villosum*), and the cooking process involves boiling for about 40 minutes then frying in onions and oil with optional addition of milk or cream [17]. Musotsi (2019) recommended that the best practices for vegetable preparation methods to preserve micronutrients should involve rapid cooking methods such as stir frying or steaming rather than boiling and intensive frying methods [18]. The recipes may be different in different regions of East Africa. However, this information about the different recipes is not documented and therefore this study could help determine the various amaranth-based food practices in different regions of Kenya and Tanzania, where amaranth consumption is considered common.

The objective of this study was to assess consumer awareness, attitudes, and utilization practices of amaranth in Kenya and Tanzania. The study is guided by the following research questions:

- Do consumers have different preferences for different amaranth?
- What are the utilization practices of amaranth?
- What challenges, beliefs, or attitudes increase or decrease amaranth utilization?

The information from this study can be used for promotion strategies and develop behavior change communication strategies to increase amaranth consumption and promote better nutrition practices.

## 2. Methodology

### 2.1. Study Areas

**Figure 1:**
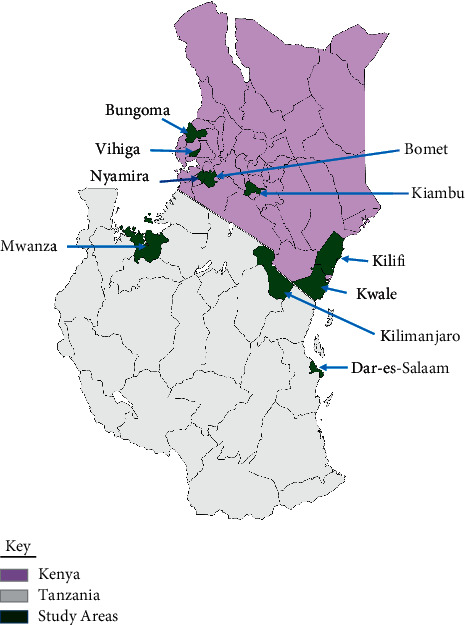
Study areas.

A total of ten study target sites including seven counties in Kenya and three regions in Tanzania were purposively selected (Figure 1), based on the leaf amaranth production volumes as documented by Horticultural Crops Directorate (HCD) in Kenya [19] and by different research reports in Tanzania [20, 21]. The high production areas also translate to high consumption areas. The counties in Kenya included Kiambu, Bungoma, Vihiga, Nyamira, Bomet, Kilifi, and Kwale, while the regions in Tanzania included Kilimanjaro, Dar-es-Salaam, and Mwanza. Two subcounties/districts were then selected purposively from the counties on the basis of their utilization of amaranth.

### 2.2. Study Participants

The study collected qualitative data from 20 Focus Group Discussions (FGDs) and 20 Key Informant Interviews (KIIs) in the ten target sites with two FGD and two KII conducted in each site.

The FGD of each study site included 8–12 community members comprising female household members from growing areas of amaranth vegetable. Female members were preferred since they are more familiar with food preparation and utilize amaranth. Recruitment of FGD participants was done purposively, with the help of county and regional officers in the areas. Key informants were purposively selected, comprising agricultural and nutrition officers who have first-hand knowledge of utilization practices in the various areas.

The criteria used in this study to select members for FGD were female/male farmers who grow and consume African Indigenous Vegetables, female/male agriculture and nutrition officers, female members of households, age above 18 from households that cook and consume AIVs including amaranth, and consent to participate in the study.

### 2.3. Ethical Considerations

Ethical clearance was sought from Jomo Kenyatta University of Agriculture and Technology Ethical Review Committee and from the WorldVeg Institutional Biosafety and Research Ethics Committee (IBREC). A research permit was also sought from the National Commission for Science, Technology and Innovation (NACOSTI). Permission was also sought from various local government authorities before undertaking the survey. Written informed consent was obtained from each participant who took part in FGD and KII. All documents including participant information sheets, consent forms, and FGD and KII guides were translated to Kiswahili. During the FGD, consent was provided by project member to all participants upon the study information; all participants were provided with a detailed study information sheet and thereafter requested to sign a consent form. Confidentiality of their data was assured and they were also assured that the data will solely be used for the study. Participation in the study was purely voluntary and all respondents were at liberty to withdraw at any time.

### 2.4. Data Collection and Analysis

Data was collected by qualitative method following set guidelines for Focus Group Discussions and Key Informant Interviews [22–24]. A total of 14 FGDs were conducted in seven counties of Kenya and 6 FGDs in three regions of Tanzania. A total of 20 Key Informant Interviews were also conducted in Kenya and Tanzania. Data collection was done between October and November 2020 in Kenya and in March-April 2021 in Tanzania. For both the FGDs and KIIs, the interviews were conducted at the locations and times that were convenient to the interviewees, with some FGDs being conducted at volunteer farmers' homes, while some including all KIIs were conducted at the subcounty and district offices. The interviews were facilitated by one researcher at any given time and supported by others for note taking and administration.

Upon administration of the informed consent, discussions and interviews were facilitated using prewritten FGD and KII guidelines (Supplementary documents (available here)). The information collected included indigenous vegetables consumed and their sources, methods of preparation, utilization, possible preservation practices, and the beliefs around amaranth vegetable. Both methods took between 30 minutes and 1.5 hours per study. All interviews were audio recorded and complemented by written short notes.

Qualitative data from the FGDs and KIIs was transcribed and translated and then coded to variable categories. Common themes were then established and clustered in a pattern order to clarify variables predicting concepts. The themes were in line with the study objectives. Inferences were then made from the data under each theme and conclusions drawn.

## 3. Results and Discussion

### 3.1. Preferred Species and Their Sources

Several African Indigenous Vegetables were reported to be consumed in the various regions of Kenya and Tanzania. From the results of the survey, consumption differs with regions, counties, and even communities in the different parts of East Africa. The common AIVs include amaranth (*Amaranthus* spp.), nightshade (*Solanum scabrum/villosum*), spider plant (*Cleome gynandra*), cowpea leaves (*Vigna unguiculata*), jute mallow (*Corchorus olitorius*), slender leaf (*Crotalaria brevidens*), pumpkin leaves (*Cucurbita moschata*), Ethiopian kale (*Brassica carinata*), vine spinach (*Basella alba*), bitter lettuce (*Launaea cornuta*), moringa (*Moringa oleifera*), *Asystasia gangetica,* wandering jew (*Tradescantia zebrina*), and black jack (*Bidens pilosa*).

Amaranth was among the most preferred vegetables in most areas where the study was done. In about 90% of the FGDs, amaranth was mentioned among the three preferred indigenous vegetable and was also the most preferred in all areas of Tanzania as well as in coastal and central counties of Kenya (Kilifi, Kwale, and Kiambu). High preference for amaranth in other areas of Africa has been reported [25]. It can be suggested that it may indeed be the most common in Africa. This high preference for amaranth compared to other AIVs has been attributed to its taste and availability of numerous species everywhere [15]. About seven major species of amaranth were reported to be known: *A. dubius*, *A. blitum*, *A. hypochondriacus*, *A. lividus*, *A. cruentus*, red amaranth (*A. hybridus*), and *A. spinosus*. However, some of these species are not used as food.

**Figure 2:**
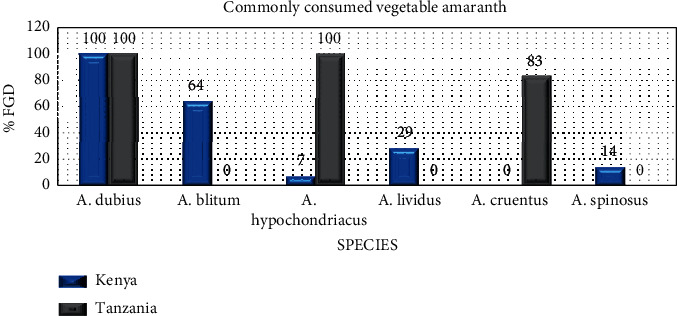
Common amaranth vegetables in Kenya and Tanzania.

**Figure 3:**
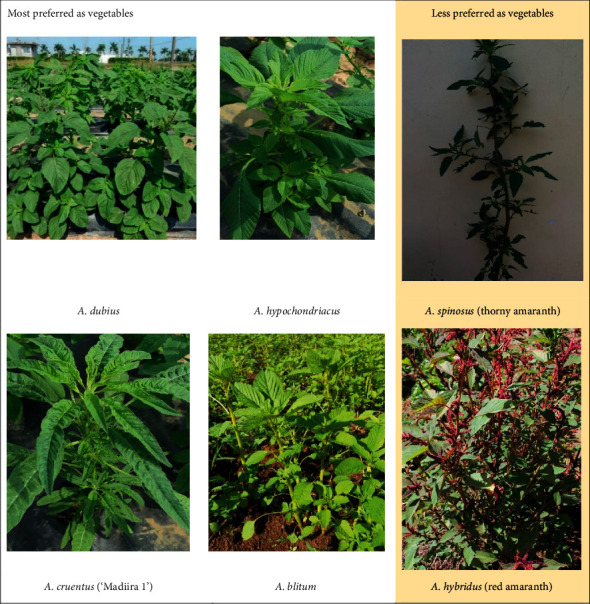
Pictures of the common amaranth species.

The respondents were familiar with different amaranth species. However, some species are more preferred for use as food, owing to their taste. The most preferred species of amaranth in Kenya were *A. dubius* and *A*. *blitum*, while *A. dubius* and *A. hypochondriacus* were most commonly used in Tanzania (Figure 2). Local taxonomy for amaranth differed widely, as some species had more than one name. Aside from the local names, leaf size and color of stem and leaves are other common characteristics that are used to differentiate the different amaranth species (Figure 3). In parts of Western Kenya and areas near the rift valley, the most preferred species have local name which are in the local languages, such as *A. dubius* (Mchicha-Swahili, Terere-Kikuyu, Ododo-Luo, Tsimboga-Luhya, Emboga-Kisii, Chelwanda-Kalenjin, Mchicha bwasi-Tanzania), *A*. *hypochondriacus* (Soisoi-Luo, Dodo-Kisii, Mchicha lishe-Tanzania), and *A. blitum* (Logatsi-Mijikenda, Livogoi-Luhya, Emboga Nyerere-Kisii, Mborochik-Kalenjin, Mchicha pwene-Tanzania). *A. lividus* was also among those reported to be consumed in Bomet and Nyamira counties. However, it is not planted and only harvested from the wild. An *A. cruentus* variety, “Madiira 1,” also has a growing preference in Tanzania. This is a newly released variety with peculiar morphology, such as long curly leaves, which has given it the nickname *mchicha bangi* as it is likened to the *cannabis* plant.

Another species of amaranth which was known but not considered for eating was *A. spinosus*, which was not preferred because of its thorns. This brings out the regional difference in preference for species, as *A. spinosus* has been reported to be among the most preferred species in South Africa [15]. *A. hybridus* (red amaranth) is also not preferred because of its deep purple soup that it produces when cooked. The red amaranth was also reported to cause stomach upset in some people. In Nyamira County of Kenya, the red amaranth was also associated with certain beliefs which hinders its consumption. “*That red one even if you find in your farm, you should pluck and throw it away,*” said an FGD respondent in Nyamira. These two amaranth species are therefore not planted and only grow as weeds. In certain areas such as Mwanza region of Tanzania, these two species were reported to have some medicinal uses.

In all the areas, the main sources of amaranth were own farm production and buying from local markets (Table 1). More farmers are venturing in the production of amaranth because it needs less inputs, has short growth period, is fairly resistant to drought, and is attacked by fewer pests, and the market demand is rising. This shows an improving trend from earlier reports which indicated that the vegetables are semicultivated and are mostly collected from the wild [26, 27]. In all the discussions in both Kenya and Tanzania, it was reported that at least 30% of community members grow amaranth for either sale, home consumption, or both. Commercial production is also practiced by some individuals in the different communities, and the varieties for this are based on preferences. In central and coastal Kenya, *A. dubius* is grown in large scale, while in Western Kenya, *A. blitum* is common for commercial production. In Tanzania, *A. hypochondriacus* is the most common for large scale production. It is planted and uprooted after 21 days for sale in markets. During rainy seasons, people rarely buy the vegetables as they are available in plenty, even appearing as weeds in their farms. During such seasons, the vegetables can also be obtained from neighbors for free. Physical appearance including tenderness and freshness was the main attribute considered by consumers when buying the vegetables, while a few consider pesticide use.

### 3.2. Preparation and Consumption

Amaranth, like other green leafy vegetables, is cooked before consumption. Cooking is done to improve palatability, texture, and taste; eliminate potential pathogens; and neutralize poisonous or irritating components [28]. The general procedure before cooking involves destalking, washing, draining, and chopping, which is optional. The cooking methods for amaranth vary with consumers, and the most common cooking practices as reported by the respondents include boiling then frying, boiling only, frying/sauteing only, steaming, and fermenting (Table 2). From this study, it was clearly noted that a great number of respondents considered taste rather than nutrition quality, yet cooking is known to cause significant changes in nutrition of vegetables.

Amaranth vegetables were prepared either on their own or in combination with other vegetables, such as cowpeas, spider plant, bitter lettuce, nightshade, and other foods such as beans, meat, and small fish (*Omena*). The reasons given for mixing with other vegetables were to reduce bitterness of vegetables such as spider plant, to improve texture in cowpeas, and to improve flavor of nightshade. In other foods, the mixing was done to save the time for cooking. In some cases, mixing was also done with more than one vegetable to increase the amount during low vegetable seasons. “*In dry seasons, one may have to harvest all the available types and mix together to get enough portion for the whole family,*” said a key informant in Kilimanjaro, Tanzania. Added ingredients vary with regions and include oil, onions, tomatoes, carrots, coconut cream/milk, cow milk/cream, peanut paste, African lye, and salt. Amaranth vegetables are mostly eaten with maize meal (*ugali*), rice, bananas, and potatoes in both Kenya and Tanzania. Generally, about 30% of the population eat amaranth as a main dish, while most eat it as side dish.

Consumption of amaranth and other indigenous vegetables was reported to be on the rise in Kenya and Tanzania. A study done in South Africa reported a contrary situation, indicating that consumption and usage of amaranth are on the decline in the region [29]. The respondents, however, noted that the younger people in their household still prefer other foods over the vegetables. A similar report was given in a study conducted in part of Kenya, where mothers reported that their young ones consume very low amounts of amaranth and other traditional vegetables in their diet [30]. Another study also reports that the rural elderly were more likely to accept and consume amaranth and other indigenous vegetables due to their experience in AIV preparation, cooking, consumption, and other uses [31, 32]. Even though most people consume amaranth frequently, not for its nutritional attributes but because it is cheap and available or because of circumstance related to poverty, there has been notable change in cooking from the traditional methods. The traditional methods involved long boiling time extending to hours, coupled with fermentation for several days [17, 33–35]. It was noted by the key informants that training and advise on cooking are taken seriously and there have been some changes in attitudes. “*We have been advising our clients, the old, those with children and HIV patients to incorporate the amaranth in their foods,*” said a nutrition key informant in Nyamira County. However, some individuals still prefer the old cooking habits. “*My mother's vegetable was much better than my wife's, the old methods were very good and that is why I do not have ulcers. I beseech people to go back to the old cooking methods,*” said an FGD respondent in Bungoma.

### 3.3. Medicinal and Alternative Uses of Amaranth

Amaranth and all the other indigenous leafy vegetables are believed to have nutritional and medicinal properties. Majority of respondents believed that the rise in consumption of amaranth in their regions has greatly contributed to fewer cases of some illnesses such as eye problems and cases of anemia. “*Nowadays there are no drugs (supplements) we are given for blood issues, we eat vegetables,*” said a respondent in Kilifi. This could be due to its richness in iron and beta-carotene among other micronutrients [36]. This corroborates with the results of a study in Tanzania, which associated the consumption of indigenous vegetables with micronutrient adequacy, and the absence of indigenous vegetables in an area can be an indication of micronutrient deficiencies especially in women of child-bearing age [37]. Amaranth seeds can be used in porridge flour. This is mainly for the dual-purpose types that also produce edible grains. In some communities, lactating mothers and people with low blood are advised to boil amaranth and drink the soup to boost their blood. In some cases, raw amaranth leaves were blended into juice or pounded to get the extract. Such juice was used as an appetizer. In Kilifi County, amaranth was mixed with jute mallow (teleza) and taken to relieve constipation. This can be attributed to the high contents of soluble and insoluble fibre in amaranth [38, 39]. In Bomet County, amaranth was used as a traditional detoxifier; the leaves are boiled with pepper and the soup is taken hot; it causes a lot of sweating which was reported to remove toxins from the body. “*You just boil it with chilies, drink the soup and sit under a tree. Sweat will just be flowing and it cleans your body,*” said an FGD respondent in Bomet. The sweat induction and fever reduction by amaranth have been reported to be due to its soporific and febrifuge effects [40]. In the same region of Bomet, Mborochik (*A. blitum*) is mixed with another herb and the extract used as relief for menstrual pain.

Ash from a burnt amaranth stalk was reported to stop bleeding. Thorny amaranth was also used as medicine to cure mouth wounds in children and ulcers, as reported in one FGD in Mwanza, Tanzania. The whole plant is harvested, dried, and burned to char (not to white ash). This is then ground to powder, mixed with a little salt, and licked, without mixing with water. Other studies have reported wound healing and anti-inflammatory properties in amaranth [41, 42]. *A. dubius* has been reported to control inflammation [43], while *A. spinosus* was reported to control oedema [44]. Additional finding in this study was that red amaranth can be charred and ground to cure coughs and tonsils. The charred powder from red amaranth can also be used to cure external wounds. Red amaranth was used to make *ugoro* (tobacco snuff), charred, mixed with wood ash, and dissolved in water. This was then mixed with ground tobacco to make it stronger/bitter. Apart from their use as food and medicine, amaranth leaves, grains, and stalk also have other nonfood uses. Excess produce, especially during rainy seasons, can be used as animal feed (cattle and chicken) to make organic manure and the stalks can also be used for firewood. Although farmers feed amaranth to animals solely to reduce loses, use of the leaves and grains of amaranth has been reported to lead to better health and production in several farm animals [45].

### 3.4. Beliefs and Taboos

Some positive beliefs contribute to increased consumption of amaranth. For instance, amaranth is believed to “add blood” in the body, contribute to good eyesight, eliminate marasmus in children, boost milk production for breast feeding mothers, and help in cleaning the kidney. “*We rarely have low HB case here because we encourage the consumption of amaranth, we rarely even give iron supplements,*” said a nutrition key informant in Nyamira County. “*If you eat a lot of amaranth, there's no day you will be told you do not have enough blood,*” said a respondent in Kilifi. Despite its availability and nutritional attributes, amaranth vegetable still remains underexploited, and this can partly be attributed to certain negative beliefs about the consumption of the vegetable or use of some species.

Amaranth is a food for the poor. While amaranth may be considered as worth a superfood [40], the vegetable being poor man's food as reported during the study is an indication that the information on the nutritional importance of the vegetable is still needed. “*We still have a hard time convincing people in the rural areas that these vegetables are better than exotic, the urban people have quite changed,*” said a key informant in Vihiga County. At a Focus Group Discussion in Kwale County in Kenya, the community members stated that they eat a lot of amaranths because they are poor and cannot afford more precious foods. “*Many eat amaranth because they are poor, if you give me a lot of money now, I will not run to buy amaranth, you do not need money to get amaranth. Very few eat it for nutritional benefits,*” said a respondent in Kwale County. In some FGDs in Tanzania, it was also reported that eating amaranth as the main dish is a sign of poverty, and the rich can only use it as a side dish. Similar study reported that a few members of the community associated the vegetables with low class people and poor households [46]. Most respondents reported that they would not freely serve amaranth meal to visitors, as it can show that they lacked a proper food to serve. “*If you serve amaranth vegetables only to a visitor, you will be seen as if you do not have anything,*” said respondents in Mwanza. In a study on knowledge of indigenous vegetables in three counties in Kenya, it was reported that most respondents disagreed that the vegetables are poor people's food, food for older generation, or old-fashioned food [47]. The study also reported high positive attitudes towards AIVs in Busia, Nyamira, and Machakos Counties of Kenya.

The first sprouts of amaranth at the start of rainy season are believed to cause stomach upset in some individuals. This was reported in 5 FGDs in Kenya. While this was not reported in Tanzania, a case of red amaranth causing bloody diarrhea was mentioned. In western Kenya, there is some skepticism about growing and consuming the grain/dual purpose types of amaranths, for example, *A. hypochondriacus*. This is because when this type was introduced in the region, seeds were given to HIV/AIDS patients. Therefore, the grain/dual purpose amaranths are still being stigmatized. Red amaranth (*A. hybridus*) is not consumed in certain parts of Kenya, because it is believed to causes fights/wrangles in homes when cooked in the home (*mboga ya fitina/troublesome vegetable*). This belief was very evident in the two FGDs that were conducted in Nyamira County in Kenya. “*That vegetable, if you just cook and eat in your house, troubles and quarrels will just start and you will not even understand why,*” said a respondent in Nyamira. The key informants also confirmed that this belief exists, but no one really understands how the vegetable is related to household wrangles.

Some taboos related to harvesting of amaranth were also reported. For instance, it is a taboo for a woman who gave birth and whose baby came out legs first to harvest the vegetables as the vegetables will dry up. Another one was that if a baby develops upper teeth first before the lower teeth, then the mother cannot harvest vegetables as they will dry up mysteriously. Certain members of the community were not allowed to harvest vegetables; they were believed to have some curse that destroys vegetable farms. It was also believed that some harvesting containers affect growth of the vegetables; the vegetables will not grow well if harvested vegetables are put in a tray (*uteo*) or wrapped in a cloth. “*Since the tray is for threshing, if you use it for harvesting, you are chasing away the vegetables,*” said an FGD respondent in Kilifi. It was also believed that vegetables should not be harvested by pinching but only by using a knife to harvest. It was also believed that allowing different people in the vegetables garden can cause drying; one should harvest his/her own vegetables, even if they are for somebody else to use.

### 3.5. Preservation

In most study areas, preservation of amaranth was not practiced. The major reason given by the respondents for not preserving is that the vegetable is readily available in most seasons of the year. In Nyamira County in Kenya, the respondents reported that a few people dried the vegetables solely to send them to their relatives living in overseas countries. The absence or limited practice of preservation of the vegetable leads to a lot of wastage due to postharvest losses, especially during seasons of vegetable abundance. In a few areas, there is recent training on preservation. “*We have a project where we are trying to build capacities on vegetable preservation. Currently very few are doing preservation,*” said a key informant in Bungoma. For most vegetable traders, the usual practice, which has also been reported in other studies, is sprinkling of water to maintain the freshness as long as possible [48]. The lack of preservation further leads to low consumption of the vegetables in dry seasons, when the vegetables availability gets very low and the prices are high.

### 3.6. Challenges and Ways of Improving Production and Consumption of Amaranth Vegetables

Most of the challenges put across during this study were production-related, with a few consumption-related challenges (Table 3). These challenges were mentioned in almost all the FGDs and KIIs, an indication that these challenges cut across all the counties and regions in Kenya and Tanzania.

Majority of the respondents identified lack of quality seed of desirable varieties as one of the main challenges restricting cultivation of amaranth. In Vihiga County, Kenya, several respondents noted that the seed of *A. blitum*, which is the most preferred in the region, is very scarce and expensive when found. “*I think we lost touch with seed producers and multipliers, so this specie is slowly disappearing; but it is the one that is most preferred by our farmers,*” said a key informant in Vihiga, Kenya. This is aggravated by the fact that this vegetable is mostly harvested by uprooting and very rarely left to produce seeds. In Dar-Es-Salaam in Tanzania, it was also noted that not many seed companies have taken up the production and supply of indigenous vegetable seeds including amaranth. Lack of market during rainy seasons when the vegetables are abundant is coupled with very low prices during these seasons. For this reason, some farmers have been demoralized and have abandoned the production of these vegetables. Training on reduction of these losses is important in dealing with this problem. The challenge of pests and diseases especially in dry seasons has led some farmers to use a lot of pesticides on these vegetables, and, in most instance, the postharvest interval is not adhered to. The farmers therefore require training on how to produce these vegetables organically and on proper use of pesticides. Lack of water for some people during dry seasons affects both production and consumption. Farmers either have to stop production or get alternative water sources. The production is also affected by pests. Prices of vegetables then go up, which affects availability and affordability, leading to reduced consumption. Despite the effect of drought on the vegetables, it was noted that the vegetables would still be available in dry seasons. Similar findings have also been reported in other parts of Africa [4, 25], insinuating the drought tolerance of the vegetables. Other consumption-related challenges were stomach upset for some people caused by red amaranth, hence hindering its consumption. “*This red one if you eat a lot, you'll really have diarrhea,*” said a respondent in Kiambu County. Some respondents also reported that they got stomach upsets when they consumed a lot of the vegetables at the beginning the of rainy seasons. Some people around the urban centres produce the vegetables using dirty water, creating fear of consumption among consumers in these areas. “*When your vegetables have large leaves, buyers can suspect you have done sewage farming, even when you haven't,*” said a key informant in Kiambu County. Most key informants in Kenyan counties reported that changes in local government structures had destabilized most nutrition-related extension works. It was further reported that in some cases, crucial agrinutrition department, which synergized agriculture and community nutrition, had been removed. This had created a big challenge in agrinutrition information transfer to community members. “*As crops department, we used to work with nutritionists, but the county governments came with different priorities, and our nutritionists were taken to health department; we hoped to have home economics, but they are busy with other areas in meat and fisheries,*” said agricultural key informant in Kenya.

Besides these challenges, preference for exotic vegetables caused underproduction and underutilization of amaranth vegetables. This was coupled with social changes that led to loss of traditional food knowledge, as well as the transition of the dietary lifestyles to fast foods, especially in younger generation who preferred sugary, fatty, and salty tastes of snacks and fast foods [40, 49, 50]. Novel preparation methods that encourage consumption of amaranth based foods are therefore recommended.

Training on the nutritional importance of the vegetable amaranths was the most requested information in all the areas where the study was carried out. “*Some individuals harvest the vegetables and leave them in the sun, not knowing the effect of that on the nutrients,*” said a key informant in Dar-es-Salaam, Tanzania. Face-to-face training coupled with demonstrations was reported as the most preferred mode of receiving information in all the FGSs and KIIs.

## 4. Conclusion

From this study, preference for indigenous vegetables was found to differ with region as well as individuals. Amaranth was shown to be a common vegetable in Kenya and Tanzania. Although many species of amaranth are known to people, *A. dubius* and *A. blitum* were the most common for vegetable consumption in Kenya, while *A. dubius* and *A. hypochondriacus* were most common in Tanzania. Red Amaranth and the spiny amaranth were less preferred in all the regions. The preference for species was mostly based on taste and availability. Consumption of amaranth vegetables was generally reported to be increasing, with many people growing it for both sale and home consumption. Some people also still wait for it to self-replicate as a weed, while others buy it from the markets. Cooking methods varied and included boiling and frying. Most people considered taste rather than nutrition quality when cooking; hence, some methods led to reduced nutritional value. Most people also consumed the amaranth merely because it was cheap and available or because of circumstance and would not eat the vegetable if they had a better option. The major constraints were low availability in dry seasons, pests, lack of quality seed in some places, and short shelf life. The study pointed out some cooking practices that destroy nutrients as well as some negative beliefs and attitudes in a few areas. Training on nutritional importance and conservation of the vegetables is needed.

## Figures and Tables

**Table 1:** Summary of responses from survey in Kenya and Tanzania.

County/region		KM	BG	VG	NY	BT	KF	KL	KR	DR	MZ	(%)
Sources	Grown	✓	✓	✓	✓	✓	✓	✓	✓	✓	✓	100
	Bought	✓	✓	⊗	✓	✓	✓	✓	✓	✓	✓	90
	Found wild	✓	✓	✓	✓	✓	✓	✓	⊗	⊗	⊗	70

Amaranth as main ingredient		✓	⊗	⊗	⊗	✓	✓	✓	✓	✓	✓	70
Amaranth as minor ingredient		✓	✓	✓	✓	✓	✓	✓	⊗	⊗	⊗	70
Preservation		⊗	⊗	⊗	✓	⊗	⊗	⊗	⊗	✓	⊗	20

Other uses	Medicinal	✓	✓	✓	✓	✓	✓	✓	⊗	⊗	✓	80
	Animal feed	✓	✓	⊗	⊗	⊗	⊗	⊗	✓	✓	✓	50

Beliefs	Adds blood	✓	⊗	⊗	⊗	⊗	✓	✓	✓	✓	✓	60
	Good for eyes	⊗	⊗	⊗	⊗	⊗	⊗	⊗	✓	✓	✓	30
	Sign of poverty	⊗	⊗	⊗	⊗	✓	⊗	✓	⊗	⊗	✓	30
	Causes stomach upset	✓	✓	✓	⊗	✓	⊗	⊗	⊗	⊗	⊗	40

Taboos		⊗	⊗	⊗	✓	✓	✓	⊗	⊗	⊗	⊗	30

KM, Kiambu, BG, Bungoma, VG, Vihiga, NY, Nyamira, BT, Bomet, KF, Kilifi, KL, Kwale, KR, Kilimanjaro, DR, Dar-es-Salaam, MZ, Mwanza, TT, total.

**Table 2:** Common amaranth cooking methods in Kenya and Tanzania.

Recipe name	Method	Areas where it is practiced
Boiling only	Wash vegetables, layer the cooking pot with banana leaves to avoid burning of the vegetables, arrange the unchopped vegetables sprinkling a little salt between the layers, boil without turning (for about 1 hour) until the water dries up to prevent steam from escaping	Bungoma, Vihiga, Bomet
	Old recipe: no washing, boiled for about one hour, add salt to taste	Nyamira
	Boil in water for 20 minutes, add salt to taste (milk or cream can also be added)	Nyamira, Bomet, Kilifi, Kwale

Boiling then frying	Boil for 10–20 minutes, drain the water, and then fry in oil, onions, and tomatoes. Cook while stirring for about 10 minutes	Bungoma, Vihiga, Nyamira, Bomet, Kwale, Kilifi, Mwanza
Steaming	Wash vegetables and place in sufuria, add chopped onions, tomatoes, and salt and steam for 10–15 minutes	Kwale, Kilimanjaro, Dar-es-Salaam, Mwanza

Frying/sauteing	Fry onions and tomatoes in oil, add chopped amaranth, and cook while stirring for about 10 minutes	Kiambu, Bungoma, Vihiga, Bomet, Kilifi, Kwale, Kilimanjaro, Mwanza
	Wash, dry in the sun to drain water, then fry in oil, onions, and tomatoes	Nyamira, Bomet, Kwale

Fermenting	Boil for 20 minutes, add salt to taste, heat once a day while adding milk each time for 2 to 3 days (mostly done in combination with other vegetables)	Bungoma

Mixed with other vegetables	Wash all the vegetables, boil together for about 30 minutes, fry in oil, onions, and tomatoes (cowpeas, spider plant, nightshade)	Bungoma, Nyamira, Bomet, Kilifi, Kwale, Kilimanjaro

Amaranth with coconut oil	Place in sufuria, add coconut milk and tiny wild cherry tomatoes, cook for 15 minutes, and serve	Kilifi

Gunjamato	Boil amaranth, add maize flour to make a mixture of ugali and vegetable	Kwale

With bhajia	Mix chopped amaranth leaves with gram flour, make into bhajia dough, and fry	Kwale, Kilimanjaro,

**Table 3:** Challenges and possible solutions.

Challenge	Solutions suggested by respondents
Lack of quality seed of preferred varieties	Researchers to work with seed companies on seed availability and qualities such as productivity, pest resistance, flood, and drought resistance
Lack of markets in seasons of abundance	Improve market structure to ease marketing
	Train on vegetable preservation and value addition
Low vegetable volumes in dry seasons	Train on water conservation and kitchen gardening
Pests and diseases	Training on pest control and safe pesticide use
Amaranth causes stomach upset in some people	Training on proper preparation and cooking
Vegetable safety concerns	Training on good agricultural practices and safe pesticide use
Little information on nutritional value and nutrient conservation in the vegetable	Return agrinutrition department in local governments

## Data Availability

The qualitative data used to support the findings of this study are available from the corresponding author upon request.

## Supplementary Materials

Supplementary Materials The participant information sheet, consent form for participants, and guiding questions used in the Focus Group Discussions and Key Informant Interviews.

## References

[1] Abukutsa-Onyango M. O. (2011). *Researching African Indigenous Fruits and Vegetables – Why?*.

[2] Matenge S. T. P., van Der Merwe D., de Beer H., Bosman M. J. C., Kruger A. (2012). Consumers’ beliefs on indigenous and traditional foods and acceptance of products made with cow pea leaves. *African Journal of Agricultural Research*.

[3] Blas A., Garrido A., Unver O., Willaarts B. (2019). A comparison of the Mediterranean diet and current food consumption patterns in Spain from a nutritional and water perspective. *The Science of the Total Environment*.

[4] Dlamini V., Viljoen A. T. (2020). Investigating knowledge on indigenous green leafy vegetables amongst rural women in Eswatini. *Journal of Consumer Sciences*.

[5] Karanja D., Okoko N., Kiptarus E. (2012). *Promoting Farmer Led Seed Enterprises of African Indigenous Vegetables to Boost Household Incomes and Nutrition in Kenya and Tanzania*.

[6] Krause H., Fabe A., Grote U. (2019). Welfare and food security effects of commercializing African indigenous vegetables in Kenya. *Cogent Food Agric*.

[7] Lin J.-T., Liu S.-C., Shen Y.-C., Yang D.-J. (2011). Comparison of various preparation methods for determination of organic acids in fruit vinegars with a simple ion-exclusion liquid chromatography. *Food Analytical Methods*.

[8] Biesalski H. K. (2013). *Hidden Hunger. (Der Verborgene Hunger)*.

[9] Nyonje W. A., Makokha A. O., Abukutsa-Onyango M. O. (2014). Anti-nutrient, phytochemical and antiradical evaluation of 10 amaranth (*Amaranthus* spp.) varieties before and after flowering. *Journal of Agricultural Science*.

[10] Akaneme F. I., Ani G. O. (2013). Morphological assessment of genetic variability among accessions of *Amaranthus hybridus*. *World Applied Sciences Journal*.

[11] Erum S., Naeemullah M., Masood S., Qayyum A., Rabbani M. A. (2012). Genetic divergence in *Amaranthus* collected from Pakistan. *Journal of Animal and Plant Sciences*.

[12] Croft M. M., Marshall M. I., Weller S. C. (2014). Consumers’ preference for quality in three African indigenous vegetables in Western Kenya. *Journal of Development and Agricultural Economics*.

[13] Gido E. O., Ayuya O. I., Owuor G., Bokelmann W. (2017). Consumption intensity of leafy African indigenous vegetables: towards enhancing nutritional security in rural and urban dwellers in Kenya. *Agricultural and Food Economics*.

[14] Kansiime M. K., Ochieng J., Kessy R., Karanja D., Romney D., Afari-Sefa V. (2018). Changing knowledge and perceptions of African indigenous vegetables: the role of community-based nutritional outreach. *Development in Practice*.

[15] Mncwango N. C., Mavengahama S., Ntuli N. R., Jaarsveld C. M. V. A. N. (2020). Diversity, consumption dynamics and ethnomedical claims of traditional leafy vegetables consumed by a rural community in the KwaMbonambi area, Northern KwaZulu-Natal, South Africa. *Biodiversitas*.

[16] Hossain A., Khatun M. A., Islam M., Huque R. (2017). Enhancement of antioxidant quality of green leafy vegetables upon different cooking method. *Preventive Nutrition and Food Science*.

[17] Musotsi A. A., Abukutsa-Onyango M., Makokha A. (2017). Changing food consumption habits: a case of African indigenous vegetables for food and nutrition security in Kakamega county, western Kenya. *African Journal of Horticultural Science*.

[18] Musotsi A., Makokha A., Abukutsa-Onyango M., Kilonzi S. M. (2019). Quantitative changes of ascorbic acid and beta carotene in African nightshade (*S olanum* nigrum) and spider plant (*Cleome gynandra*) due to traditional cooking methods used in Western Kenya. *Journal of Food Science and Nutrition Research*.

[20] URT (2017). Annual agriculture sample survey. https://nbs.go.tz/nbs/takwimu/Agriculture/2016_17_AASS_report.pdf.

[21] Ochieng J., Schreinemachers P., Ogada M., Dinssa F. F., Barnos W., Mndiga H. (2019). Adoption of improved amaranth varieties and good agricultural practices in East Africa. *Land Use Policy*.

[22] Dzino-Silajdzic V. (2018). *Focus Group Discussions, Practical Guide*.

[23] World Bank (2020). *Understanding People’s Perspectives on Identification: A Qualitative Research Toolkit*.

[24] van Eeuwijk P., Angehrn Z. (2017). *How to Conduct a Focus Group Discussion (FGD) Methodological Manual*.

[25] Omotayo A. O., Ndhlovu P. T., Tshwene S. C., Aremu A. O. (2020). Utilization pattern of indigenous and naturalized plants among some selected rural households of North West Province, South Africa. *Plants*.

[26] Chipungahelo M. S. (2015). Knowledge sharing strategies on traditional vegetables for supporting food security in Kilosa district, Tanzania. *Library Review*.

[27] Jansen van Rensburg W. S., Zulu N. L., Gerano A. S., Adebola P. O. (2015). Seed production of African leafy vegetables: some experiences. *Acta Horticulturae*.

[28] Odendo M., Ndinya-Omboko C., Merchant E. V., Minyatta-Onyango E. (2020). Do preferences for attributes of African indigenous vegetables recipes vary between men and women? a case from Western Kenya. *Journal of Medicinal Plants*.

[29] Maseko I., Id T. M., Tesfay S. (2017). African leafy vegetables: a review of status, production and utilization in South Africa. *Sustainability*.

[30] Gewa C. A., Onyango A. C., Obondo Angano F. (2019). Mothers’ beliefs about indigenous and traditional food affordability, availability and taste are significant predictors of indigenous and traditional food consumption among mothers and young children in rural Kenya. *Public Health Nutrition*.

[31] Ayanwale A. B., Amusan C. A., Adeyemo V. A., Oyedele D. J. (2016). Analysis of household demand for underutilized indigenous vegetables. *International Journal of Vegetable Science*.

[32] Gido E. O., Ayuya O. I., Owuor G., Bokelmann W. (2017). Consumer acceptance of leafy African indigenous vegetables: comparison between rural and urban dwellers. *International Journal of Vegetable Science*.

[33] Musotsi A., Sigot A., Abukutsa-Onyango M. African indigenous vegetables recipe documentation and their role in food security.

[34] Wakhanu J. A., Kimiywe J., Hudson N. (2016). Oxalate levels in selected african indigenous vegetable recipes from the lake victoria basin, Kenya. *International Journal of Environmental Research*.

[35] Wafula E. N., Franz C. M. A. P., Rohn S., Huch M., Mathara B., Trierweiler J. M. M. (2016). Fermentation of African indigenous leafy vegetables to lower post-harvest losses, maintain quality and increase product safety. *African Journal of Horticultural Science*.

[36] Ashraf M. Y., Ashraf M., Ozturk M. (2018). Underutilized vegetables: a tool to address nutritional issues, poverty reduction and food security. *Global Perspectives on Underutilized Crops, *.

[37] Conti M. V., De Giuseppe R., Monti M. C. (2012). Indigenous vegetables: a sustainable approach to improve micronutrient adequacy in Tanzanian women of childbearing age. *European Journal of Nutrition*.

[38] Chidozie Ogwu M. (2020). Value of *Amaranthus* (L.) species in Nigeria. *Nutritional Value of Amaranth*.

[39] Sarker U., Oba S. (2019). Protein, dietary fiber, minerals, antioxidant pigments and phytochemicals, and antioxidant activity in selected red morph Amaranthus leafy vegetable. *PLoS One*.

[40] Olusanya R., Unathi K., Nomali N., Chinsamy M. (2021). Underutilization versus nutritional-nutraceutical potential of the Amaranthus food plant: a mini-review. *Applied Sciences*.

[41] Moyo S. M., Mavumengwana V., Kayitesi E. (2018). Effects of cooking and drying on phenolic compounds and antioxidant activity of African green leafy vegetables. *Food Reviews International*.

[42] Vithya S., Jayshree N. (2017). Phytochemical analysis and in vitro anticancer study of ethanolic extract of leaves of *Amaranthus cruentus* Linn. against colon cancer cell line (Hct-116). *World Journal of Pharmacy and Pharmaceutical Sciences*.

[43] Tufts H. R., Harris C. S., Bukania Z. N., Johns T. (2015). Antioxidant and anti-inflammatory activities of Kenyan leafy green vegetables, wild fruits, and medicinal plants with potential relevance for Kwashiorkor. *Evidence-Based Complementary and Alternative Medicine*.

[44] Olajide O. A., Ogunleye B. R., Erinle T. O. (2004). Anti-inflammatory properties of *Amaranthus spinosus* leaf extract. *Pharmaceutical Biology*.

[45] Manyelo T. G., Sebola N. A., van Rensburg E. J., Mabelebele M. (2020). The probable use of genus Amaranthus as feed material for monogastric animals. *Animals: An Open Access Journal from MDPI*.

[46] Chacha J. S., Laswai H. S. (2020). Traditional practices and consumer habits regarding consumption of underutilised vegetables in Kilimanjaro and morogoro regions, Tanzania. *International Journal of Food Science*.

[47] Ntawuruhunga D., Affognon H. D., Fiaboe K. K. M., Abukutsa-onyango M. O. (2020). Farmers’ knowledge, attitudes and practices (KAP) on production of African indigenous vegetables in Kenya. *International Journal of Tropical Insect Science*.

[48] Gogo E. O., Opiyo A., Ulrichs C., Huyskens-Keil S. (2018). Loss of African indigenous leafy vegetables along the supply chain. *International Journal of Vegetable Science*.

[49] Taleni V., Goduka N. Perceptions and use of indigenous leafy vegetables (ILVs) for nutritional value: a case study in mantusini community, Eastern cape province, South Africa.

[50] Mayekiso A., Taruvinga A., Mushunje A. (2017). Perceptions and determinants of smallholder farmers’ participation in the production of indigenous leafy vegetables: the case of Coffee Bay, Eastern Cape province of South Africa. *African Journal of Science, Technology, Innovation and Development*.

