# Supplementary material for: Exploring the Status of Preference, Utilization Practices, and Challenges to Consumption of Amaranth in Kenya and Tanzania
Source: J Nutr Metab. 2022 Jun 18;2022:2240724. doi: 10.1155/2022/2240724 (PMC9233608; doi:10.1155/2022/2240724)
Supplement: Supplementary Materials — The participant information sheet, consent form for participants, and guiding questions used in the Focus Group Discussions and Key Informant Interviews. [file 2240724.f1.docx]

# Supplemental Documents

# Participant Information Sheet

**Consumer Survey on Amaranth Vegetable Utilization Practices**

***Introduction***

We are a team of researchers from Jomo Kenyatta University of Agriculture and Technology (JKUAT) in collaboration with the World Vegetable Centre (WordVeg/AVRDC). We are carrying out research on the utilization and consumption practices of amaranth vegetables across parts of Kenya and Tanzania. This is part of a PhD research project conducted by ***Winnie Nyonje*** at JKUAT. This research seeks to study Amaranth food preparation methods and recipes and develop a behavior change strategy for improved nutrition in East Africa.

***Procedure:*** If you accept to participate in this research, we will conduct a focused group discussion, where we will be discussing about amaranth vegetable utilization in this area. The data collected will be used provide information on the various cooking methods and utilization practices of amaranth vegetables in East Africa.

***Benefits:*** The benefits of your participation in this study include contributing to knowledge on amaranth production and consumption practices in East Africa, as well as any ways to improve its consumption. Your knowledge, concerns and aspirations will be compiled and shared with other researchers, educators and decision makers to determine next steps in improving food and nutrition status in the communities. There is no monetary benefits.

***Risks/discomfort:*** No known risk is expected from participating in this research. We would like to ask you, however, not to tell us anything that is sensitive and might damage your reputation, financial standing or present any unacceptable risk to you.

***Confidentiality:*** Your study data will be handled as confidentially as possible. If results of this study are published or presented, individual names, quotes, photographs and other personally identifiable information will not be used unless you give explicit permission for this.

***Rights:*** Your participation in this research is **completely voluntary**. You are free to decline to take part in the study and you can also decide to withdraw at any moment without a reason. If you choose to decline or withdraw from the interview, there will be no penalty to you or loss of benefits to which you are otherwise entitled.

This study is conducted by Winnie Nyonje, from the department of Human Nutrition Sciences, JKUAT. For any questions, contact Winnie Nyonje on +254723363401, e-mail: [winnienyonje@yahoo.com](mailto:winnienyonje@yahoo.com)

For further concerns, contact:

***Prof. Mary Abukutsa – study supervisor***

Jomo Kenyatta University of Agriculture and Technology (JKUAT)

P.O. Box 62000-00200, Nairobi.

This research is approved by the Jomo Kenyatta University of Agriculture and Technology (JKUAT) ethical review board and WorldVeg Institutional Biosafety and Research Ethics Committee (IBREC)

#

# Consent Form

**Consumer Survey on Amaranth Vegetable Utilization Practices**

I understand the purpose of the research and the language that is being used for the interview. I therefore voluntarily and freely agree to participate. I understand that the information I give will be purely for the purposes of this research. My questions and concerns have been addressed well and I am free to withdraw from the interview at any point without a reason and there will be no consequences.

Participant’s Name …………………………………………………………..…………………………

Signature …………………………………… Date ……………………….

Should you have any question or problems contact Winnie Nyonje:

***Department, Human Nutrition Sciences, JKUAT***

Phone: +254723363401

E-mail: [winnienyonje@yahoo.com](mailto:winnienyonje@yahoo.com)

Or

***Prof. Mary Abukutsa***

Jomo Kenyatta University of Agriculture and Technology (JKUAT)

P.O. Box 62000-00200, Nairobi.

# Guidelines for Focus Group Discussion and Key Informant Interview on Amaranth Utilization Practices

**FGD Guide for Amaranth Vegetable Utilization Practices**

Country …………….……… County/ Region ……………… Sub-county/ District ………………

**Discussion questions**

1. Which African indigenous vegetables (AIVs) produced and consumed in this area and how do they compare to other vegetable?
2. What are the common varieties of amaranth grown and consumed in this area? Are there varieties that are more preferred or disliked and why?
3. Where do people in this area obtain amaranth for consumption, and how are the amaranth vegetable prepared and cooked?
4. What do people think about the consumption of amaranth and other AIVs vegetables in this area? Are there beliefs and taboos associated with the vegetables? If so, which ones.
5. What is the general perception of people in this area about preservation of amaranth vegetables and other indigenous vegetables?
6. What are the constraints/challenges affecting the production and utilization of amaranth in the community?
7. How can production and consumption of amaranth vegetables be improved at the community and household level; and what kinds of information would prompt you to include more amaranth and other indigenous vegetables in your meals?
8. How do you prefer to receive information about amaranth? (Such as demonstrations by experts, discussion with neighbors, through training, printed materials, radio, TV, text messages, social media, etc.)

**KII Guide for Amaranth Vegetable Utilization Practices**

Date of Interview ……………..… Country …………….…. County/ Region ……….…….………

Time Start: ……………..……………………. Time stop: ………………….………………..

Interviewer Name ……………………………………………………………………………………

Name of Respondent ……………………..………. Position of Respondent ………………..……

Organization ……………………………………………..…………………………………………..

**Interview Questions**

1. Which indigenous vegetables are mostly produced in this area?
2. Are there indigenous vegetables that are more preferred than others? Which ones and why?
3. Are there different varieties/types of amaranth vegetables produced in the area? What characteristics are used to differentiate them?
4. What has been the production and consumption trends of amaranth vegetables and other indigenous vegetables in the area, increasing or decreasing and why?
5. Are they produced mainly for sale or home consumption?
6. How are the amaranth vegetables utilized? (as vegetables or medicine or other uses)
7. Please describe the general cooking method of vegetable amaranth in the area
8. Do the community members preserve amaranth and other vegetables? If yes what are the methods of preservation
9. What are the constraints/challenges to production and consumption of amaranth vegetables in the area?
10. Are there any beliefs or taboos related to vegetables amaranth in this area? If yes, explain
11. How can the production of amaranth and other indigenous vegetables be improved in your community?
